# Structure and development of flowers and inflorescences in Peraceae and Euphorbiaceae and the evolution of pseudanthia in Malpighiales

**DOI:** 10.1371/journal.pone.0203954

**Published:** 2018-10-03

**Authors:** Karina Bertechine Gagliardi, Inês Cordeiro, Diego Demarco

**Affiliations:** 1 Departamento de Botânica, Instituto de Biociências, Universidade de São Paulo, São Paulo, São Paulo, Brazil; 2 Centro de Pesquisa em Plantas Vasculares, Núcleo de Pesquisa Curadoria do Herbário, Instituto de Botânica, São Paulo, São Paulo, Brazil; Indiana University Bloomington, UNITED STATES

## Abstract

Pseudanthia are reduced and compact inflorescences which apparently had independent evolution in Euphorbiaceae and Peraceae within Malpighiales. In order to analyze the hypothesis that the different pseudanthia found in Malpighiales have non-homologous developmental steps, we studied the inflorescence and flower development in the three Malpighiales genera that present this type of inflorescence–*Dalechampia* (Acalyphoideae/Euphorbiaceae), *Euphorbia* (Euphorbioideae/Euphorbiaceae), and *Pera* (Peraceae)–and compared them to that of *Joannesia* (Crotonoideae/Euphorbiaceae), which does not present a pseudanthium. Inflorescences and flowers were analyzed using light microscopy and scanning electron microscopy. *Dalechampia* and *Euphorbia* have protogynic bisexual pseudanthia, with unisexual perianthed flowers in *Dalechampia*, and achlamydeous flowers in *Euphorbia*. *Pera* has unisexual pseudanthia and the male flowers have a vestigial calyx and the female flowers are achlamydeous. *Joannesia* flowers are very distinct when compared to the pseudanthia flowers, as they are composed of all the whorls and there is no reduction. In the early stages of development, the first structures to be formed in the pseudanthia are the different series of bracts, including outer, involucral and involucel bracts. The floral primordia are initiated almost simultaneously with the involucre. Although the different morphology, the early inflorescence followed the same branching pattern in all studied genera, and the number and elongation of the branches were affected by the early female flower development in the terminal position. We suggest that the different pseudanthia evolved via process of floral whorl reduction and reorganization of flowers in the inflorescence axes, especially the position of female and male flowers and elongation or shortening of the branches. The sex of the terminal flower is a developmental key, *i*.*e*., the protogynic development deeply affects the pseudanthia growth, reducing the ramification and elongation of the axes.

## Introduction

Pseudanthia are compact inflorescences which occur in more than 40 angiosperm families and can be interpreted as an aggregation of diminutive flowers that composes an attraction unit [1] and resemble a single flower [2–4].

Within Malpighiales, pseudanthia are found in *Euphorbia*, the cyathium, which is a synapomorphy of the genus and the subject of several recent studies [5–8]. However, pseudanthia also occur in *Dalechampia* and *Pera* [5,9,10], which were both previously placed in Acalyphoideae-Euphorbiaceae. Currently, *Dalechampia* still belongs to the subfamily Acalyphoideae, but *Pera* is included in the family Peraceae, sister to Euphorbiaceae + Rafflesiaceae and apparently there have been independent origins of pseudanthial inflorescences within Malpighiales [5,11,12].

The floral diversity and complexity of pseudanthia in Malpighiales has led to several studies on the ontogeny of their flowers and inflorescences aiming to understand the developmental steps that culminated in such different morphologies. Classen-Bockhoff [1] emphasized that an ontogenetical abbreviation likely played an essential role in the origin and elaboration of pseudanthia.

Froebe and Magin [13] described the pseudanthia elaboration after studying *Dalechampia* morphology and ontogeny and stated that huge complexity in the organization of flowers determines variation in the inflorescence patterns. Prenner and Rudall [5] and Prenner *et al*. [14] performed a detailed ontogenetic analysis of the Euphorbieae subtribes and suggested that the cyathium evolved from a strong condensation of branches and flowers. Later, Prenner *et al*. [8] also investigated the boundary between flower and inflorescence and stated that genes associated with floral development are also expressed in the inflorescence apex during pseudanthia development.

Recently, Endress *et al*. [15] analyzed several floral structures in Malpighiales and revealed the emergence of several new subclades following modification of the phylogenetic trees. This urged the prompt undertaking of studies on floral development to enable the structure, biology, and evolution of these groups to be elucidated. For example, Euphorbiaceae *s*. *str*. contains almost 200 genera and still requires floral studies on the gynoecium structure, especially concerning the ovule integuments and its development.

Although there is an effort to understand floral evolution in the Malpighiales, little is known about the ontogenetic factors that led to the occurrence of pseudanthia in the order. Thus, for this study, we selected the three pseudanthia types found in Malpighiales (*Dalechampia*, Acalyphoideae/Euphorbiaceae; *Euphorbia*, Euphorbioideae/Euphorbiaceae, and *Pera*, Peraceae). These species were chosen because each of them represents a unique type of pseudanthia in the order. Our aim is to test the hypothesis that the different pseudanthia have distinct developmental steps and have evolved due to heterochronic modifications. We also studied *Joannesia* (cymose, paniculiform inflorescence) because its flowers have no sign of reduction and they are not arranged in pseudanthia, thereby the species is used for comparison and to test the hypothesis that the pseudanthia evolution is linked to the reduction of floral whorls and inflorescences.

We performed a comparative analysis on the degrees of whorl reduction based on meristem activity and ontogenetic factors in order to understand and suggest an initial explanation for the changes in floral development that culminated in the formation of the three pseudanthia types in Malpighiales.

## Materials and methods

### Materials

Flowers and inflorescences of the following species were harvested: *Euphorbia sipolisii* N.E. Br. (K. Gagliardi & D. Demarco 5), *Dalechampia meridionalis* Müll. Arg. (K. Gagliardi & D. Demarco 8), *Pera glabrata* (Schott) Poepp. ex Baill. (K. Gagliardi & D. Demarco 7) and *Joannesia princeps* Vell. (K. Gagliardi & D. Demarco 6).

### Bright field microscopy

Inflorescences containing flowers at several developmental stages were collected at the Instituto de Biociências of Universidade de São Paulo and at the Instituto de Botânica in São Paulo. Vouchers of the species were provided and the vouchers were deposited in the herbarium of the Universidade de São Paulo (SPF). The material was fixed in formalin, acetic acid, 50% ethyl alcohol (FAA) for 24 h [16] or by buffered neutral formalin (BNF) for 48 h [17], and then stored in 70% ethyl alcohol.

Inflorescence meristems, flower buds, and anthetic and post-anthetic flowers were isolated, dehydrated in a butyl series [16], embedded in Paraplast (Fisher Healthcare, Houston, Texas, USA), and transversely and longitudinally sectioned using a Leica RM2145 rotary microtome (Leica Microsystems, Wetzlar, Germany). Serial sections around 12 μm thick were stained with astra blue and safranin [18], and mounted in Permount resin (Fisher Scientific, Pittsburgh, Pennsylvania, USA). Samples were observed and photographed using a Leica DMBL light microscope (Leica Microsystems, Wetzlar, Germany).

### SEM observations

For the ontogenetic study, additional micromorphological analyses were carried out using material fixed in FAA. After the isolation of floral parts, the material was dehydrated in an ethanol series, critical-point dried in a Balzers CPD 030 (Balzers, Liechtenstein, Germany), mounted on aluminum stubs, and sputter coated with gold using a Balzers SCD 050 (Balzers, Liechtenstein, Germany) [19]. Samples were observed and images were captured using a Zeiss DSM 940 scanning electron microscope (Carl Zeiss, Oberkochen, Germany).

## Results

### Morphology of the pseudanthia

**Fig 1:**
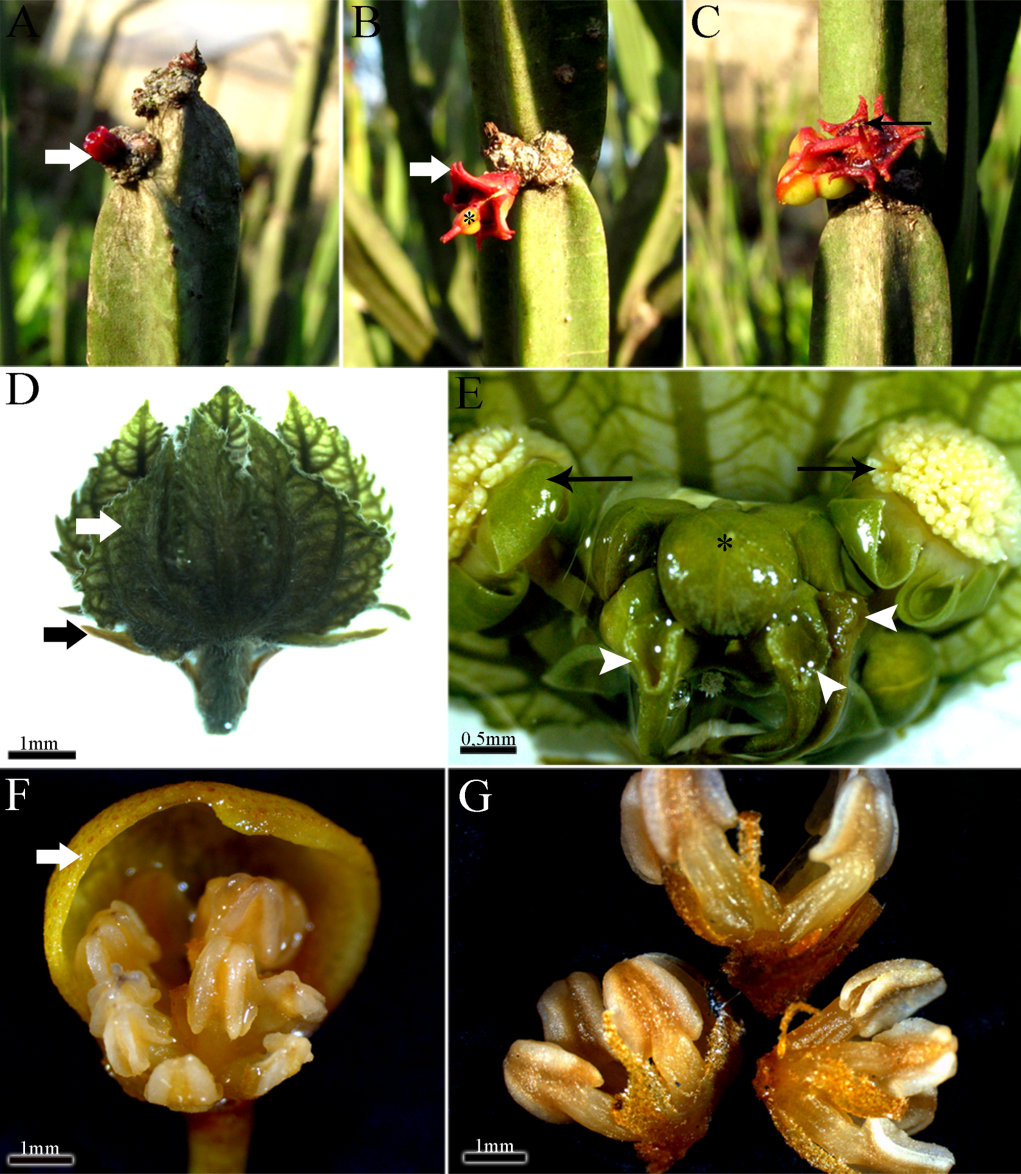
Morphology of the pseudanthia. A-C, *Euphorbia sipolisii*. Note the involucre still closed (white arrow); A-B, Young pseudanthium. Note the involucre opened (white arrow) and pistillate flower (asterisk); C, Mature pseudanthium with staminate flowers (black arrow); D-E, *Dalechampia meridionalis*. Inflorescence axis with outer bracts (black arrow) and involucral bracts closed (white arrow); E, Sub-inflorescences. Note the stigma portion (arrow heads), the terminal staminate flower (asterisk) and the lateral staminate flowers (black arrow); F-G, Pseudanthium with male flowers of *Pera glabrata*. Note the involucre (thick arrow); G, Stamens arranged in groups.

The pseudanthium of *Euphorbia* (cyathium) (Fig 1A–1C) is bisexual and composed of 20 male flowers organized in five groups of sub-inflorescences around a single and terminal female flower (Fig 1B and 1C). The pseudanthium of *Dalechampia* is bisexual and composed of 8–10 male flowers also arranged in secondary cymes and a single lateral female cyme in the proximal position (Fig 1D and 1E). *Pera* is dioecious and presents unisexual pseudanthia with the male pseudanthium with eight fertile flowers (2 stamens each) arranged in a composed cyme (Fig 1F and 1G), and the female pseudanthium has four flowers organized in a simple cyme.

All of the studied pseudanthia share several series of bracts, including: 2–4 alternate outer bracts, which are the most external bracts of the pseudanthia (Fig 1D), and the involucral bracts. These later bracts surround all the inflorescence and there are two, free and green bracts in *Dalechampia* (Fig 1D), five connate and red in *Euphorbia* (Fig 1A–1C), and two connate and yellow in *Pera* (Fig 1F). In addition, *Dalechampia* has involucel bracts (Fig 1D and 1E), which are internal to the involucral ones and closely associated with the male and female sub-inflorescences. In *Euphorbia*, the involucel bracts are exclusively subtending the male sub-inflorescences.

### Branching patterns of the inflorescences

**Fig 2:**
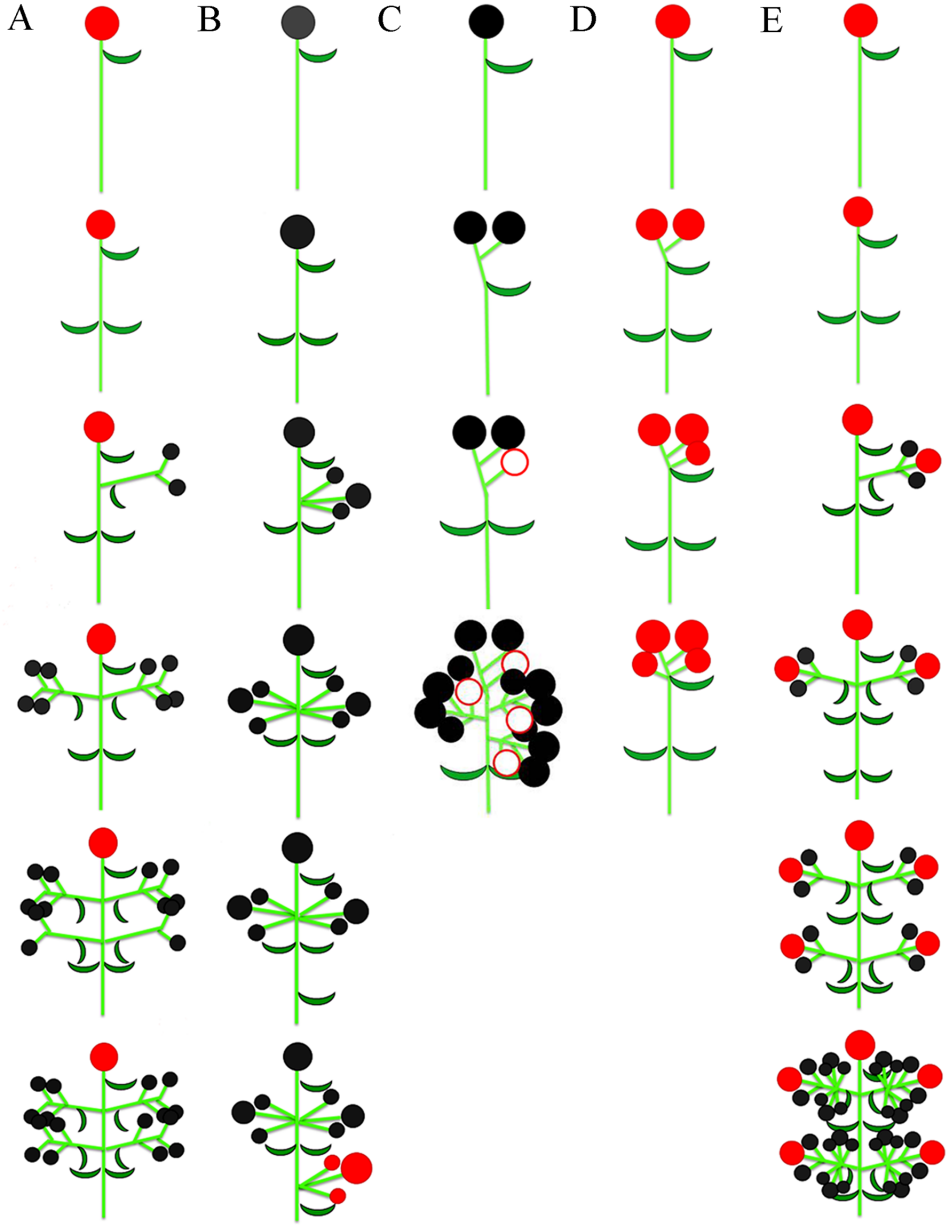
Branching and development patterns of the pseudanthia. A, *Euphorbia sipolisii*; B, *Dalechampia meridionalis*; C-D, *Pera glabrata*. C, Pseudanthium with male flowers and a pistillode; D, Pseudanthium with female flowers; E, *Joannesia princeps*. *Symbols*. Full red circle = pistillate flower; full black circle = staminate flower; empty red circle = pistillode.

The flowers of *Euphorbia* are arranged in a composed cyme (20 male flowers arranged in cymules of 4 flowers surrounding a single female flower in the centre), which is a determinate inflorescence composed of groups of cymes. This inflorescence begins the development by the female terminal flower of the main axis, followed by the ramification of five lateral axes, in which male secondary cymes are formed (Fig 2A).

The flowers of *Dalechampia* are spatially separated in a composed cyme (8–10 male flowers arranged in cymules of 2 or 3 flowers and 3 female flowers composing a cyme), and inflorescence development starts with the male terminal flower in the main axis. The ramification of three lateral branches (male sub-inflorescences), in addition to the terminal flower, composes the male portion of the inflorescence. Below this male portion, one more ramification develops, which is formed by three female flowers (Fig 2B).

The male pseudanthium of *Pera* is also a composed cyme (8 flowers arranged in cymes of 2 flowers each) with decussate branches, and starts its development with one or two flowers in the top of the main axis. These flowers will compose a cyme with the development of two other flowers. With the development of the terminal flower, the ramification of three lateral branches occurs, in which terminal flowers are formed in each branch, followed by one more female achlamydeous flower in the composition of the pseudanthia (Fig 2C). The female pseudanthium of *Pera* is also a cyme, composed of four flowers. Firstly, two terminal flowers develop and two other flowers give rise by branching of the same axis (Fig 2D).

The flowers of *Joannesia* are also arranged in a composed cyme, although this inflorescence is more ramified than the pseudanthia. Firstly, a terminal female flower develops along the main axis. Concomitantly, the ramification of several lateral branches occurs, which is followed by the development of a female terminal flower in each branch. The ramification of this secondary axis culminates in the development of two opposite male flowers, which will compose a cyme with the terminal female flower. With the development of these lateral cymes, there are two almost opposite ramifications of the lateral branches. Male cymes develop from these new dichasial branches (Fig 2E). The inflorescence of *Joannesia* have a branching pattern essentially similar to those of all pseudanthia studied here, *i*.*e*., protogynic cymes, in which the female terminal flower affects the inflorescence growth.

### Ontogeny of *Euphorbia* (Euphorbioideae)

The male flowers of *Euphorbia* are represented by a single stamen and are organized in cymes. The female flower is composed of a single gynoecium which occurs in the centre of the inflorescence and is surrounded by the male flowers.

**Fig 3:**
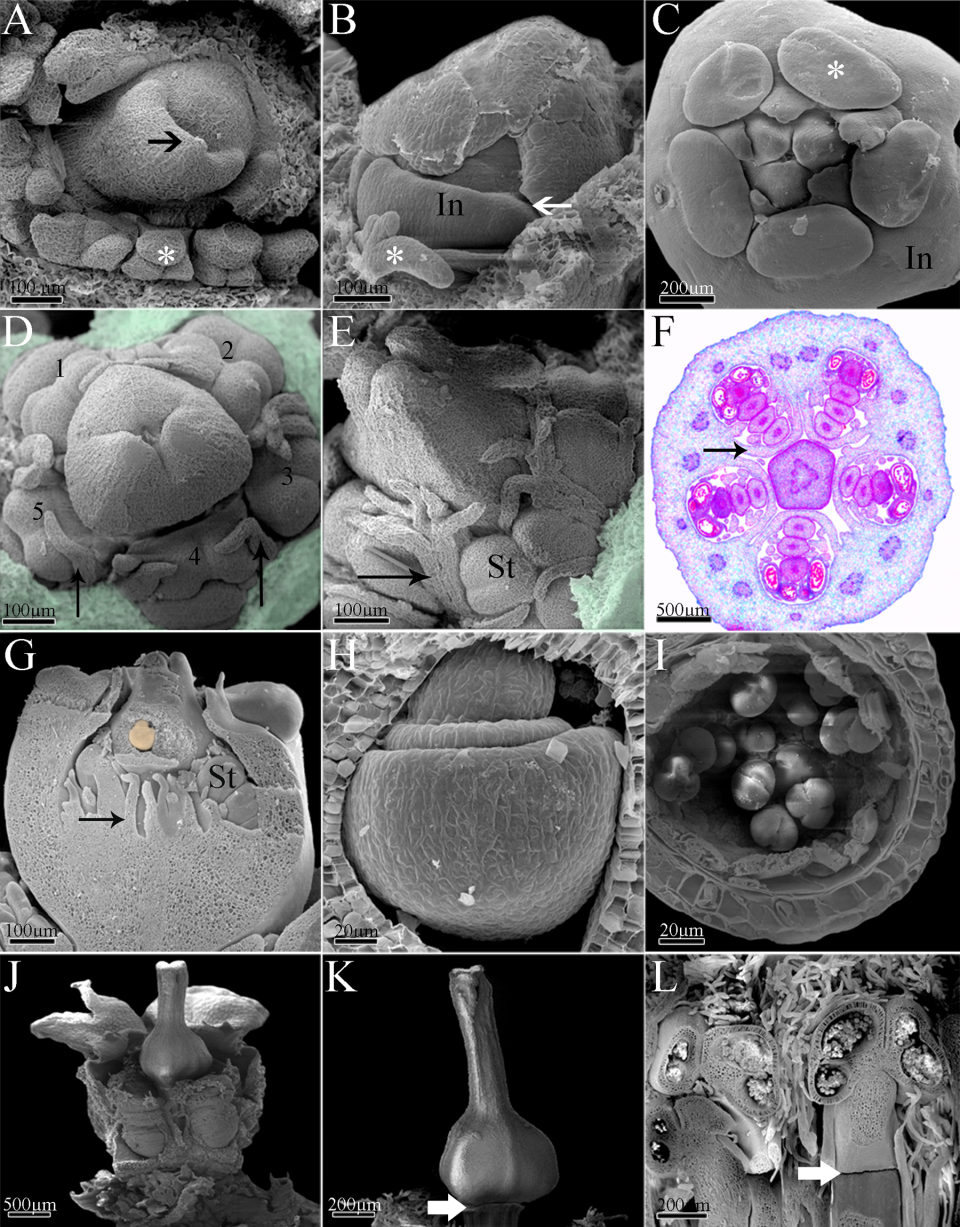
Ontogeny of *Euphorbia sipolisii*. A, Initial stage of development with outer bracts (medium arrow) and colleters (asterisk); B, Involucre development with late connation (thick arrow). Note the colleters (asterisk); C, Early pseudanthium with involucre and glands above bracts (asterisk); D, Development of the staminate secondary inflorescence (see the numbers) around the early pistillate flower. Note the involucre projection (thin arrow) and removed involucre (colorful area); E, Detail of the involucre projections (thin arrow) and developing flowers; removed involucre (colorful area); F, Light microscopy of the transversal section of the pseudanthium showing the staminate secondary inflorescence surrounded by involucre projection (thin arrow); G, Longitudinal view of the pseudanthia with developing flowers and young ovule (colored); H, Detail of the ovule with two integuments being formed; I, Detail of the whole anther wall with epidermis, endothecium, middle layer, secretory tapetum and pollen grains; J, Pseudanthium almost completely developed; K, Detail of the mature gynoecium. Note the constriction (thick arrow) between the flower pedicel and the ovary; L, Detail of the mature stamens. Note the constriction (thick arrow) between the flower pedicel and the filament of the stamen. *Abbreviations*. In, involucre; St, staminate flowers.

The first structures to be formed are the outer bracts or cyathophylls (Fig 3A), followed by the involucral bracts (Fig 3B), which are post-genitally fused forming the involucre (Fig 3B and 3C). With the elongation of the involucre, the development of glands can be observed at the apical portion of each involucral bract (Fig 3C). The involucel initiates its development fused to the internal face of the involucre (Fig 3D). The involucel bracts grow as projections of the involucre (Fig 3D and 3E) and form five chambers, which surround each male cyme (Fig 3D–3F).

Once the terminal female flower has started to develop, this is followed by the branching of the inflorescence through the formation of male sub-inflorescence primordia inside the involucral/involucel chambers (Fig 3D–3F). As well as the main inflorescence axis, the lateral axes are also determinate, and a male flower develops in the terminal position, followed by the differentiation of other flowers in a basipetal direction (Fig 3F). With the activation of the floral meristems, the ontogeny of the male flowers is observed by the development of one stamen per flower, with four flowers per cyme, for a total of 20 flowers per pseudanthium.

The female flower presents a tricarpellary syncarpous gynoecium. Initially, the base of the carpel primordia develops as one unit (synascidiate region) and forms almost the entire trilocular ovary (Fig 3D). Later, the top of the ovary and the style elongate freely (Fig 3E) and then, becomes postgenitally fused (symplicate region; Fig 3K). Finally, three bifid stigmata are formed in the top of the style (Fig 3J and 3K) and one antitropous ovule containing two integuments in each locule of the ovary (Fig 3G and 3H). Simultaneously, some male flowers already present anthers initiating the formation of pollen grains (Fig 3I). However the female flower continues to develop fast in relation to the five male inflorescences (Fig 3J). These latter have centripetal development in the cyathium (Fig 3F), which already has a complete involucre with nectaries in the upper portion (Fig 3J).

With the maturation of the flowers, the formation of a slight articulation on the pedicel of the female flower, close to the ovary base, becomes evident (Fig 3K). In the male flowers, the occurrence of this articulation establishes the boundary between the pedicel and the filament, with the formation of neither sepals nor petals in both flowers (Fig 3L). The female flower is the first to become mature. Next, the male flowers are gradually elevated above the involucre through the elongation of pedicels. The anther dehiscence may only be observed in the exserted flowers.

### Ontogeny of *Dalechampia* (Acalyphoideae)

**Fig 4:**
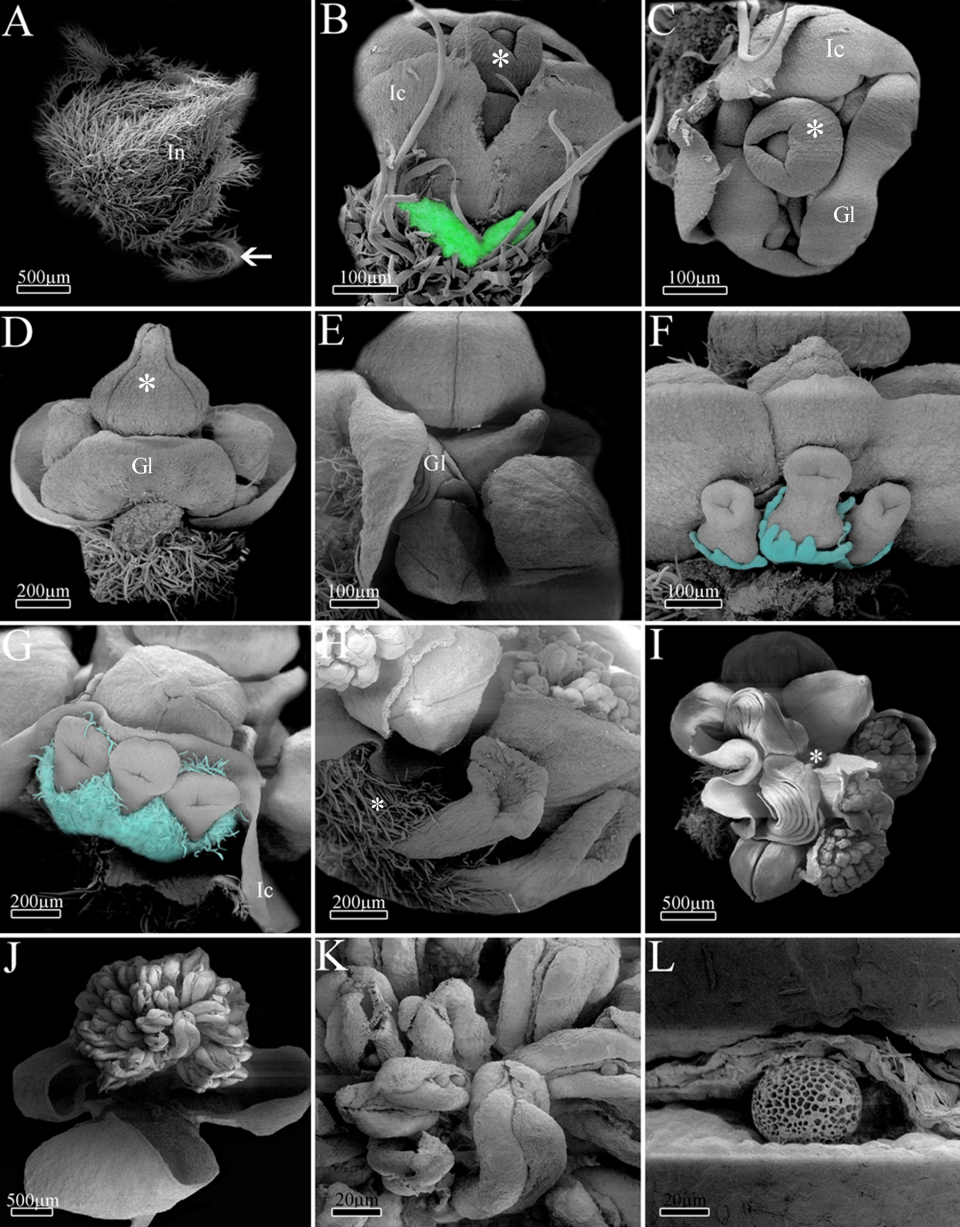
Ontogeny of *Dalechampia meridionalis*. A, Inflorescence apex with involucre completely formed and outer bracts (medium arrow); B-C, Early pseudanthium with developing involucel and flowers. Involucre was removed (colorful area); D, Terminal staminate flower with calyx completely formed (asterisk); E, Lateral view of the pseudanthium demonstrating flowers and the bracteal gland; F, Early pistillate inflorescence. Note developing calyx (colored); G-H, Mature pistillate inflorescence with ovaries covered by trichomes (asterisk); I, Upper view of the pseudanthium with mature flowers and flower buds. Terminal flower removed here (asterisk); J, Detail of the staminate anthetic flower; K, Close of mature anthers; L, Detail of pollen grain being released by longitudinal slit. *Abbreviations*. Gl, bracteal gland; In, involucre; Ic, involucel.

The first structures to be formed in *Dalechampia* are the outer bracts (Fig 4A), followed by the free involucral bracts (Fig 4A) and, most internally, by the involucel bracts (Fig 4B). During involucre and involucel elongation, it is possible to observe the initial formation and elongation of the inflorescence axis with male flower primordia (Fig 4B and 4C). The first flower to be formed is in the terminal position, followed by other male flowers in a basipetal differentiation (Fig 4C).

Following the activation of floral meristems, the ontogeny of male flowers starts with the development of a single sterile whorl, the calyx (Fig 4C), with five sepals that are almost completely fused through late connation (Fig 4D). Besides perianth, the development of filaments and anthers is also observed, with about 20 stamens per flower in asynchronous initiation. After the initial development of the male flowers, a series of bracts develops in the pseudanthium base (Fig 4C–4E). This new series is formed by the overlapping of bracts, which acquire the structure of a globoid-shaped gland in the base of the male axis (Fig 4E). After the formation of this bracteal gland, a unique lateral, basal branch will originate a female sub-inflorescence with three flowers (Fig 4F).

Initially, the female flowers present calyx with free sepals, which become fused in a later stage (Fig 4F and 4G). The syncarpous gynoecium is formed in a similar way to that of *Euphorbia*. It has a long synascidiate region in the ovary and a symplicate region from the top of ovary to the stigma (Fig 4F–4H), which is wide and peltate (Fig 4H). The female flowers develop fast in relation to the male ones, and they already have receptive stigma while male flowers remain in the bud stage (Fig 4G–4I). Most likely after the female flower pollination, the male flowers open, dispersing the pollen grains (Fig 4J–4L).

### Ontogeny of *Pera* (Peraceae)

**Fig 5:**
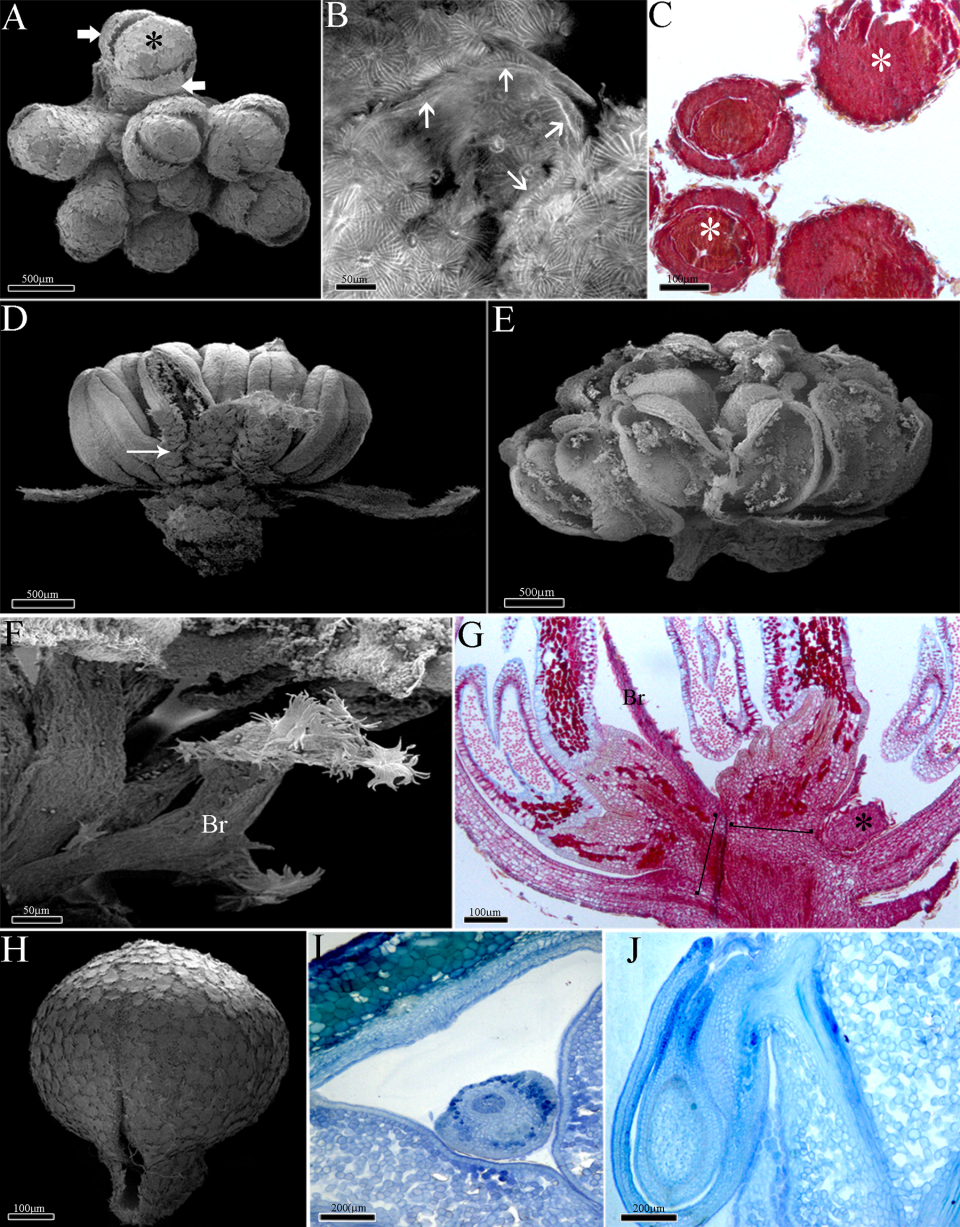
Ontogeny of *Pera glabrata*. A, Female pseudanthia primordia arranged in groups. Note the involucre (thick arrow) and meristematic region (asterisk); B, Electron confocal microscopy demonstrating involucel bracts (thin arrows); C, Light microscopy of a longitudinal section showing early male pseudanthium with developing flowers (asterisk); D, Male pseudanthium with anthetic flowers. Note the bracts associated with the flowers (thin arrow); E, Detail of male mature flowers; F, Detail of the male pseudanthia inner bract which is adjacent to the stamens; G, Light microscopy of a longitudinal section showing the male pseudanthium with pistillode (asterisk), inner bract and two staminate flowers (bars) with two stamens each; H, Female pseudanthium with involucre opened by a slight slit; I-J, Light microscopy of a detail of the female pseudanthium with developing ovule. *Abbreviations*. Br, bract; St, staminate flowers.

The first structures to be formed in the male and female pseudanthia of *Pera* are the outer bracts, which are followed by the development of the two fused involucral bracts (Fig 5A). Internally to the developing involucre, it is possible to observe the ramification of lateral inflorescence branches and the development of flower primordia (Fig 5B and 5C). The first flower to be formed is in the terminal position, followed by the formation of other flowers in a basipetal direction.

In the beginning of male pseudanthium development, it is possible to observe flowers with two stamens each, reduced filaments, and a vestigial calyx. The eight flowers are arranged in pairs (four groups of two flowers each) (Fig 5D–5F), and they are initiated asynchronically and produce pollen grains as monads (Fig 5E). The formation of a single and perianthless sterile gynoecium positioned laterally at the base of each group of male flowers is also notable, and there are therefore three or four sterile female flowers per male pseudanthium (Fig 5G). During inflorescence development the involucre of both male and female pseudanthia is not completely fused, and a narrow slit keeps the involucre opened (Fig 5H).

In the female pseudanthium, the flowers begin their development with united carpel primordia, fused in the synascidiate region of the ovary, and follow the same developmental steps of the other species. Like the sterile female flower found in the male pseudanthia, the fertile female flowers are achlamydeous and composed only of a gynoecium, which has a peltate stigma and a tricarpellary/trilocular ovary with one antitropous, bitegmic ovule per locule (Fig 5I and 5J).

### Ontogeny of *Joannesia* (Crotonoideae)

The first structure to be formed is the inflorescence bract, followed by the two bracteoles, which are associated with each floral primordium. Formation of the inflorescence is initiated by the female terminal flower at the top of the main axis. Growth continues through the development of lateral branches. Each branch differentiates a female flower in the terminal position and male flowers in a basipetal direction, as a cyme.

**Fig 6:**
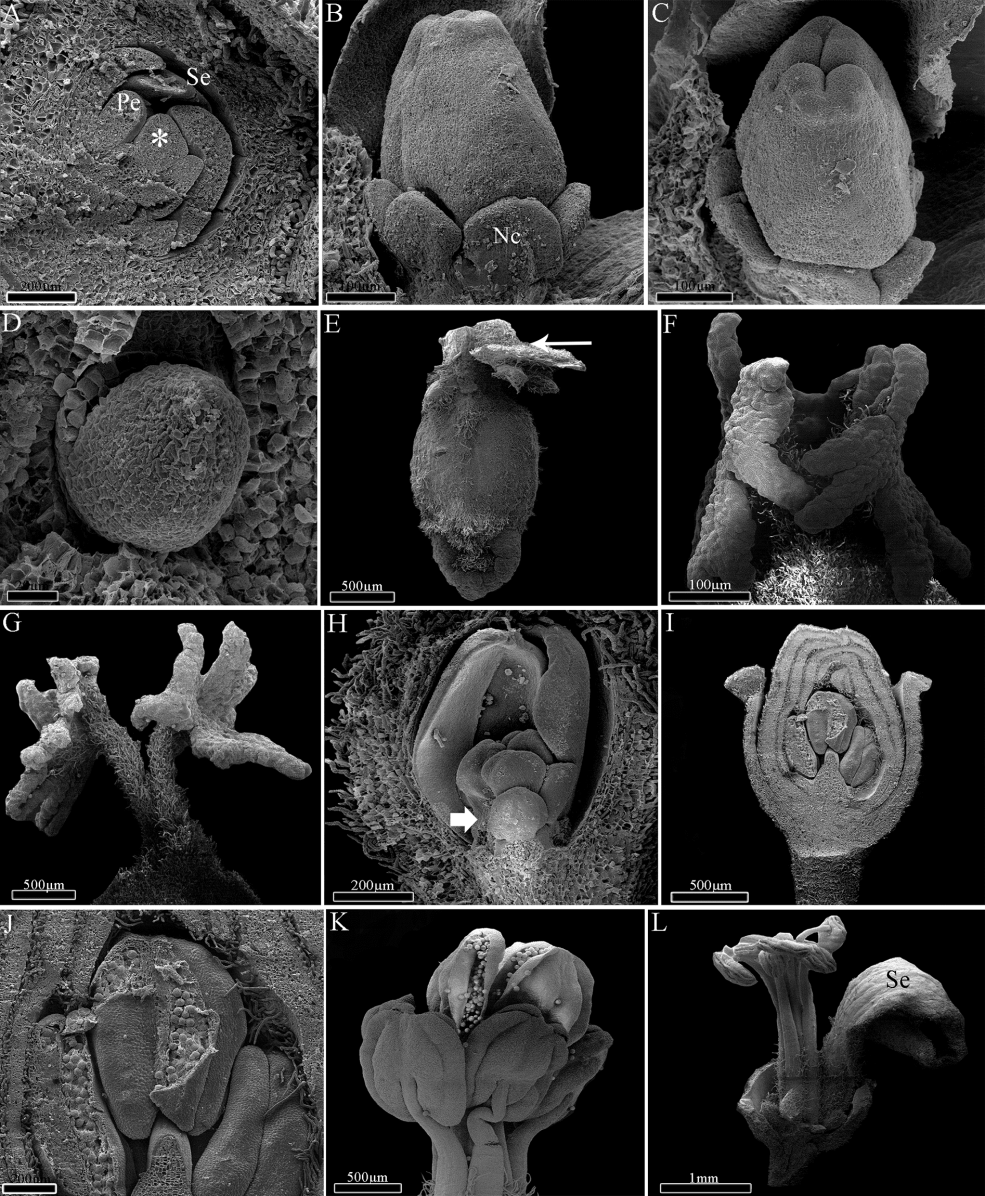
Ontogeny of *Joannesia princeps*. A, Early meristematic stage with sepals and petals primordia. Note the reproductive meristem (asterisk); B, Early gynoecium with glands on its base; C, Upper view of the carpels showing postgenital connation and developing styles; D, Detail of early ovule; E, Young gynoecium completely formed. Note the initial ramification of the styles (thin arrow); F, Detail of the branched stigma portion; G, Detail of mature styles and stigma; H, Staminate flower bud with stamens primordia. Note the developing anthers (thick arrow); I, Longitudinal view of staminate flower bud; J, Close of anthers and pollen grains; K, Detail of didynamous stamens and mature anthers; L, Senescent staminate flower. *Abbreviations*. Nc, nectary; Pe, petal; Se, sepal.

The flowers begin to differentiate with the initiation of the calyx through the development of five congenitally fused sepals, followed by five free petals primordia (Fig 6A and 6B). The petals remain free and interspersed during whole flower development. The development of calyx and corolla is similar in both female and male flowers. The female flower has syncarpous gynoecium, and the development initiates with the formation of united carpels in the synascidiate region. The gynoecium is symplicate in the top of the ovary and base of the style (Fig 6B and 6C), and the distal portion of the carpels elongate freely, giving rise to free styles and branched stigma (Fig 6E–6G). The ovary is trilocular with one antitropous ovule per locule (Fig 6D).

Synchronic growth of 6–8 stamens is observed in the male flower, with the initial, short elongation of the filaments and early expansion of the anthers (Fig 6H). The developing stamens have long filaments in a didynamous arrangement (Fig 6I and 6J). In mature stamens, the filaments are long and the anthers release pollen grains through longitudinal slits (Fig 6K). Senescent flowers present longer filaments and flattened, twisted anthers (Fig 6L).

### Comparative anatomy of flowers

#### Calyx (*Dalechampia*, *Pera*, and *Joannesia*)

**Fig 7:**
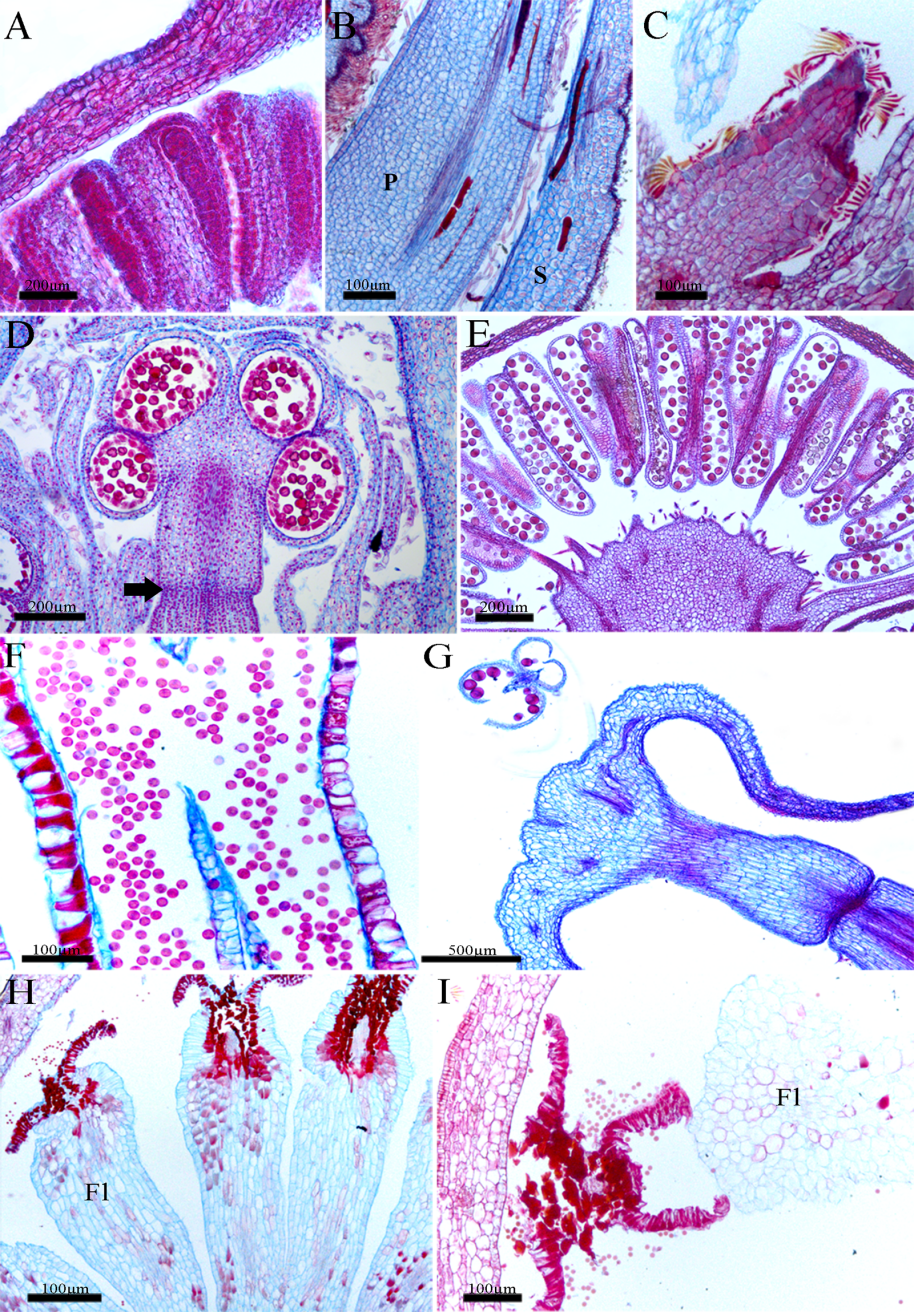
Structure of the pseudanthia: Sterile whorls and androecium. Longitudinal sections. A,E, *Dalechampia meridionalis*; B,G, *Joannesia princeps*; C,F,H-I, *Pera glabrata*. A, Detail of male flowers; B, Petal and sepal; C, pistillode;; D, *Euphorbia sipolisii*. Developing stamen with constriction region (thick arrow); E, Flower with mature stamens; F, Mature anther with pollen grains; G, Post-anthetic flowers with pollen grains being released; H-I, Mature stamens with pholiaceous aspect. *Abbreviation*. Fl, filament; P, petal; S, sepal.

The sepals are glabrous in *Dalechampia* (Fig 7A) and present indumentum in *Joannesia* and *Pera* (Fig 7B), with lepidote trichomes in the latter (Fig 7C). The epidermis is uniseriate in both faces, and is also papillose in *Joannesia* (Fig 7A and 7B). The mesophyll is homogeneous, and is composed of 6–8 layers of parenchymatic cells in *Dalechampia* (Fig 7A) and *Pera* sepals, and 8–11 layers in *Joannesia* sepals (Fig 7B). It has small, round, thin-walled cells, with collateral vascular bundles in the midrib of the species, as well as secondary and tertiary collateral vascular bundles, laticifers in *Joannesia* (Fig 7B) and idioblasts in *Pera* (Fig 7C).

#### Corolla (*Joannesia*)

The petals of *Joannesia* are anatomically similar to its sepals, with a uniseriate epidermis, slightly palisade, with an indumentum in the abaxial face and radially elongated-papillose cells from the medium region to the apex of the adaxial face. The mesophyll is homogeneous, with several layers of parenchymatic cells, which are large, visualized as different shapes (Fig 7B), with crystal idioblasts, and a midrib composed of collateral bundle. In addition, other collateral bundles constitute secondary veins.

#### Androecium (all species)

Stamens are anatomically very similar in all studied species.

The anthers are bitechae with four pollen sacs (Fig 7D and 7E). The anther walls have epidermal cells tangentially elongated and an endothecium with lignified wall thickenings. In addition, a secretory tapetum is totally consumed during the formation of pollen grains, which are arranged in monads (Fig 7D–7G). When senescent, the stamens assume a flattened aspect (Fig 7H and 7I).

#### Gynoecium (all species)

**Fig 8:**
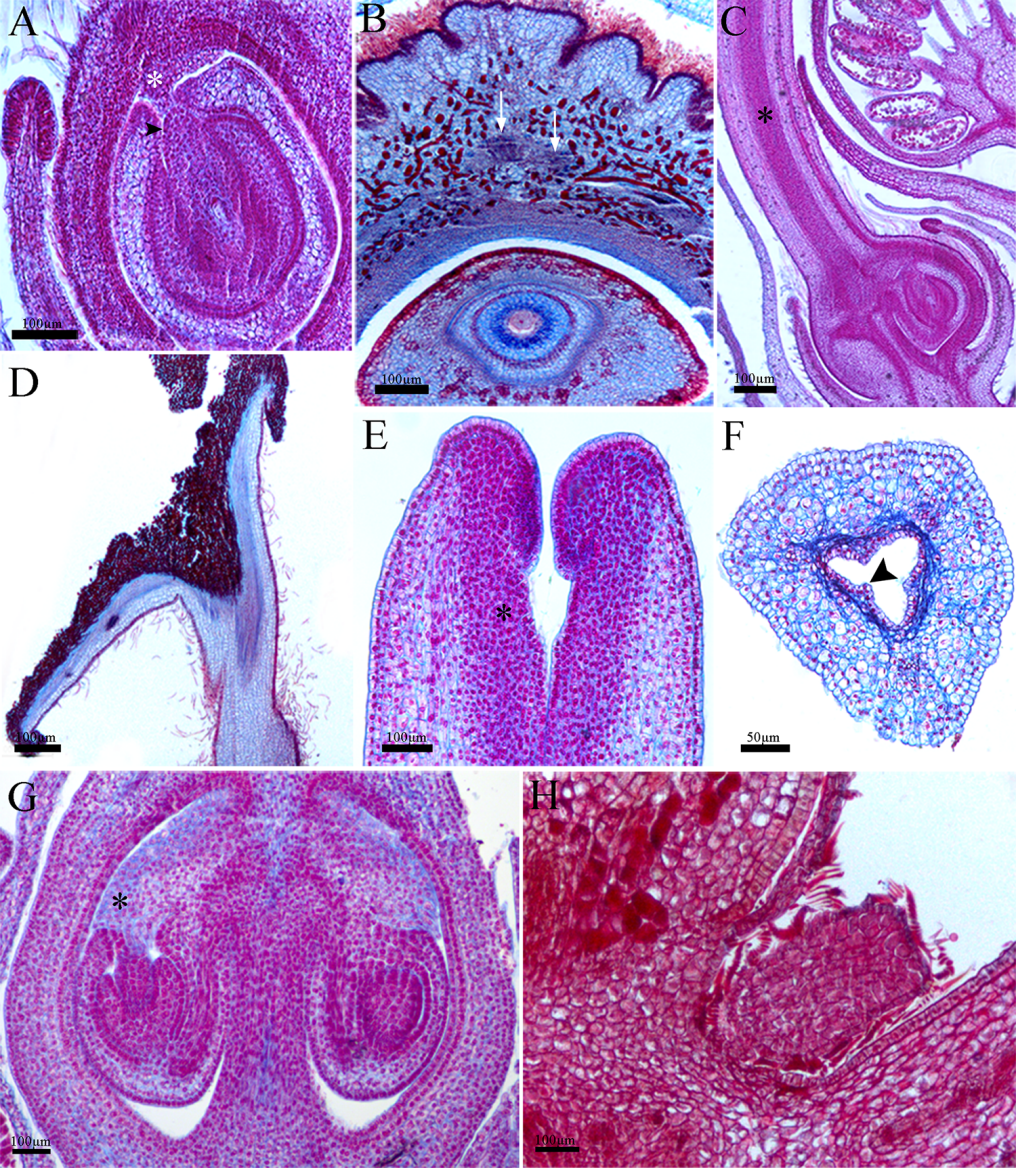
Structure of the pseudanthia: Gynoecium. A,C, *Dalechampia meridionalis*. Longitudinal section of ovary and antitropous ovule with nucellar beak (arrow-head) contacting the obturator (asterisk); B,D, *Joannesia princeps*. Cross section of the ovary and ovule. Note the vascular bundles (white arrows); C, Longitudinal section of the ovary and style. Note the transference tissue (asterisk); D, Longitudinal section of the style and stigma portion; E-G, *Euphorbia sipolisii*. Longitudinal section of style and stigma. Note the transference tissue (asterisk); F, Cross section of the hollow stigma. Note the secretory cells (arrow head); G, Longitudinal section of ovary and ovules. Note the obturator (asterisk); H, *Pera glabrata*. Longitudinal section of the sterile female flower.

The ovary presents a uniseriate outer and inner epidermis composed of isodiametric and small cells, with indumentum in *Dalechampia* and *Joannesia* (Fig 8A and 8B). This latter also presents an outer ovary epidermis with depressions and grooves along the whole wall, providing the ovary with a rugose surface (Fig 8B). The mesophyll is homogeneous in all species (Fig 8A–8C and 8G) with large dorsal and ventral collateral vascular bundles.

The styles are solid (Fig 8C and 8D), except for those of *Euphorbia*, which are hollow (Fig 8E and 8F), composed of uniseriate epidermis in both faces with a few unicellular trichomes. The central region of the solid styles is mainly parenchymatous, and a secretory duct of palisade cells is presented in the hollow style (Fig 8F). A strand of strongly stained cells can be observed in the solid style from the ovary to the stigmatic region. These cells are the pollen tube transmitting tissue and show dense aspect (Fig 8C and 8D). The stigma is hairy in *Dalechampia* (Fig 8D) and has palisade epidermis with secretory activity in the other species (Fig 8E).

The ovules have the outer integument thicker than the inner integument in all species (Fig 8A and 8G). The micropyle is composed of both integuments, with a nucellar beak protruding through the micropyle reaching the placental obturator (Fig 8A and 8G). The sterile flower of *Pera* is round and composed of an epidermis of cuboid-to-radially elongated cells with lepidote trichomes covering a parenchyma core (Fig 8H).

## Discussion

### Pseudanthia: Branching patterns and ontogeny

Considering the development of the pseudanthia studied here, and following the inflorescence classifications suggested by Weberling [2], Souza [20] and Judd *et al*. [21], the large inflorescence groups with determinate (cymose) and indeterminate (racemose), may have a different pattern not only in the occurrence of terminal flowers in some stages of development, but mainly the opening and maturation direction of the flowers, which is demonstrated in the present study.

Analysis of these definitions revealed complexity in the classifications of the pseudanthia studied here. *Euphorbia* has a determinate inflorescence, with a female terminal flower and its earlier maturation than the male flowers, the cyathium is a protogynic inflorescence. In *Dalechampia*, which also has a determinate inflorescence, the male terminal flower differentiates before the female ones; however, there is a long delay in relation to the male maturation, and the pseudanthium is protogynic. The female pseudanthium of *Pera* may be ontogenetically interpreted as a simple inflorescence, and the male one as a determinate panicle. The inflorescence of *Joannesia* may also be interpreted as a protogynic cyme, with the female flowers being the first flowers to initiate the development and complete maturation.

The determination of the inflorescences and the elongation of the branches are correlated to the bracts, which are the first structures to be formed in the early stages of development, followed by the flower primordia, as observed in the studied species. Prenner and Rudall [5] observed some notable ontogenetic differences in *Euphorbia myrsinites*, considered by the authors to be a typical example of the Euphorbiinae subtribe. In this species, the first structures to be formed are the bracts, as in the pseudanthia studied here. However, with the formation of bracts, there is the simultaneous development of the male flowers, characterizing similar ontogenetic steps to those described here for *Dalechampia* and *Pera*. In *Neoguillauminia cleopatra* (Baill.) Croizat, these authors observed that bract formation occurs almost simultaneous with the formation of male flowers, and with the formation of the central female flower. In addition, in *Dichostemma glaucescens* Pierre the bracts form almost simultaneously with pairs of stamens, and the gynoecium is the last cyathium component to be formed. Narbona *et al*. [6] analyzed the pseudanthia of *Euphorbia nicaeensis* All. and observed that after the formation of bracts, the male flowers begin to develop first, as observed here in *Dalechampia* and in the male pseudanthia of *Pera*, but not observed in *Euphorbia*.

Prenner *et al*. [8] verified almost exclusive ontogenetic factors among Euphorbiaceae inflorescences, such as *Calycopeplus paucifolius* (Klotzsch) Baill., which is considered a perfect intermediate among the pseudanthia in the family and an archetype of the cyathium [22]. In addition to the pseudanthia in the present study, Prenner *et al*. [8] found that the formation of *C*. *paucifolius* pseudanthia also begins with a series of bracts, although the development of male flowers and the single female flower occur simultaneously. This can be interpreted as a common intermediate ontogenetic step, although the female flower determinates the inflorescence growth, such as observed in the studied species.

The determination of growth is exercised by the female flower in *Euphorbia*, *Dalechampia* and *Joannesia* and by the terminal female or male flower in *Pera*. This the correlation between the development of flowers and the elongation of branches, thereby as the terminal flower (or female as mentioned above) becomes mature, the elongation of the branches stops and the different series of bracts (involucre and involucel) are formed very close to each other in a condensed inflorescence. In *Joannesia* the determination of the female flowers results in the development of bracts, bracteoles and especially in the ramification of the lateral branches.

The pseudanthia have a short axis when compared to *Joannesia*, which has long and ramified axis. This reduced pseudanthia axis can be associated with the high level of bract development and the loss of bracteoles in the flowers, such as observed in *Euphorbia*, *Dalechampia* and *Pera*. The pseudanthia bracts are highly organized for different roles, such as the involucral bracts of *Euphorbia* and *Pera*, which act to attract pollinators [23–25], and the involucel bracts of *Dalechampia*, which are closely associated with the flowers and act to protect them [4,26,27]. In addition, the bracts may be related to the floral anthesis, as reported to *Dalechampia stipulacea* Müll. Arg. [28].

In *Dalechampia*, the primordia of the male flowers are the first to start developing. This is on contrast to *Euphorbia*, in which the female flower primordia precede the development of male flowers. Although the terminal male flower starts to develop first in *Dalechampia*, there is a delay in its maturation, described by Froebe and Magin [13] as a consequent zygomorphy or disproportion. The delayed male flower development noted here is also followed by the rapid development of the female flowers, which determinates the inflorescence growth. In *Pera*, the male and female flowers develop independently because the pseudanthia are unisexual, and in both male and female *Pera* pseudanthia we observed that early development of the terminal flower determinates the reduced growth of the whole inflorescence. This fact suggests that the inflorescences have flowers in different stages of development during pseudanthia maturation, with the early and final stages of development being the most significant ontogenetic factors which are correlated to the protogynic development of the pseudanthia and *Joannesia*.

Most studies on pseudanthia of Malpighiales are related to *Euphorbia* [5–8] and *Dalechampia* [26–29] and there is no study on the development of *Pera*. Among the pseudanthia studied in Malpighiales, initiation of the floral primordia occurs in different ways, consistent with that described for the species in the present study and in the literature cited above. However, compared with individual flowers, such as those of *Joannesia*, the pseudanthia undergo fast female flower development, even when this is not the first floral morph to be formed in early stages of development.

Despite the distinct morphology of the pseudanthia studied here, the branching patterns are very similar and they have the same type of initial development. These patterns (bracts development, ramification of lateral branches and protogyny) are also the same in a plant without pseudanthium, such as *Joannesia*.

### Flowers: Ontogeny and structure

Sterile whorls were observed exclusively in the flowers of *Dalechampia* and *Joannesia*. Most studies have reported that absent or vestigial perianth occurs in pseudanthia flowers, although an articulation or constriction is often observed in reduced flowers [5–8] which marks the approximate boundary between the pedicel and the flower itself, as registered in the present study even when the perianth is completely lost.

Among the species analyzed by Prenner and Rudall [5], *E*. *myrsinites* (subtribe Euphorbiinae), which was considered derived in the phylogenetic study of Steinmann and Porter [30], presents articulation in male flowers indicating the delimitation between the filament and the pedicel. In addition, *Neoguillauminia cleopatra* (subtribe Neoguillauminiinae, phylogenetically intermediate [30] shows a vestigial and leaf-like structure close to the articulation, which is interpreted as perianth, and *Anthostema madagascariense* Baill. (subtribe Anthosteminae, phylogenetically basal) presents a well-developed perianth in the male and female flowers.

The gynoecium is the most conservative floral whorl in the species studied here, and several similarities may be highlighted such as the secretory stigmata and antitropous, crassinucellate ovules with nucellar beak. All of these characteristics are frequently registered in Malpighiales species, especially in Euphorbiaceae [31–37].

### Evolution of pseudanthia in Malpighiales

The species analyzed belong to Euphorbiaceae (Acalyphoideae, Crotonoideae and Euphorbioideae) and Peraceae. Acalyphoideae is a basal subfamily of Euphorbiaceae and clearly paraphyletic, while Crotonoideae has an intermediate phylogenetic position, without strong evidence of their monophyly. Euphorbioideae is considered to be the most derived and unique monophyletic subfamily [11]. Peraceae came from the elevation of Peroideae, Euphorbiaceae past subfamily [12,38].

The studied groups belong to phylogenetically distant genera of Malpighiales (Euphorbiaceae and Peraceae), which emphasizes that floral reduction occurred at least three times during the evolution of the order [5,11,12]. The study of *Euphorbia*, *Dalechampia*, and *Pera* demonstrates how this convergent evolution was achieved in terms of development. We observed that the different pseudanthia of Euphorbiaceae and Peraceae and the *Joannesia* inflorescence share similar developmental steps. This possible homologous branching pattern might have evolved once in the ancestral of the clade and be secondarily modified by evolutionary convergent key steps, such as floral reduction, floral re-organization in the inflorescence, internode shortening, and development of different series of bracts. Although Rafflesiaceae are now placed as sister group of Euphorbiaceae [38,39], the reduction of the inflorescence branching and the floral gigantism in that family should be related to its sapromyophilic pollination system [40] and/or to the parasitic habit. This is an initial study that indicates a possible homologous early ontogeny of the inflorescences in cyathial and non-cyathial members of the clade but new studies using a more comprehensive sampling is necessary to corroborate or refute our hypothesis.

Based on the branching patterns of the inflorescences, we classified the inflorescence of *Dalechampia* as a composed cyme, in which the male flowers are arranged along the inflorescence axis and the only lateral branch originates a female sub-inflorescence. However, Webster and Webster [41] analyzed the morphology of *Dalechampia* inflorescences and interpreted them as a terminal male pleiocasium juxtaposed by a cyme of three female flowers, surrounded by two bracts. Regardless of the interpretation of the nature of the *Dalechampia* inflorescence, it is unique among the Euphorbiaceae, and the modification of the floral structure for resin production instead nectar represents an uncommon adaptation among angiosperms [42].

Similar to *Dalechampia*, the *Euphorbia* cyathium may be considered as an inflorescence composed of a single, terminal, and central female flower in a composed cyme, which has branches of male secondary cyme inflorescences, constituting a specific determinate panicle.

The cyathium is the typical *Euphorbia* pseudanthium. Webster [10] characterized the cyathium as a pseudanthium inflorescence type, composed of a unique female flower and four or five male monochasia or dichasia, with reduced perianth or perianthless.

Recent studies have discussed the classification of this pseudanthia. Based on the results of our analysis, we agree with the hypothesis suggested by Prenner and Rudall [5], which states that the cyathium is composed of a group of male inflorescences, each one adjacent to a bract, surrounding a single terminal female flower.

The pseudanthia of *Pera* has flowers arranged inside the involucral bracts, with one in the terminal position. Currently, it is difficult to interpret pseudanthia of *Pera*, and the present literature contains no information about the ramifications and organization inside the involucre and this is the first developmental study on the flowers and pseudanthia of the genus. Based on our results, we considered these pseudanthia to be a female single cyme and a male panicle.

The inflorescence of *Joannesia* organizes its flowers in cyme groups, with the main difference between the pseudanthia established by the elongation of the lateral branches, which is related to the inflorescence meristem activity initially undetermined with a delay in the terminal flower differentiation. This slow differentiation also occurs in the lateral branches and culminates in the production of a more ramified inflorescence, although with the same development pattern.

Given the structural complexity of the pseudanthia, and the importance of floral evolutionary studies, new ontogenetic studies analyzing gene expression related to flower development could elucidate the activity of flower and inflorescence meristems on the early or late formation of flowers.

## Conclusions

We conclude that pseudanthia evolved not only from a heterochronic reduction process, but also from a rearrangement of the flowers and bracts along the inflorescence axis. With the comparative study of three pseudanthia types found in Malpighiales, represented by the genera *Euphorbia*, *Dalechampia* and *Pera* and through the comparison with *Joannesia*, we reject the hypothesis that the different pseudanthia in Malpighiales have very distinct developmental steps. We observed that all inflorescences analyzed share the same early developmental steps, which might indicate a homologous initial ontogeny in the clade. However, new studies with a more comprehensive sampling are needed to evaluate this hypothesis.

All inflorescences have protogynic maturation, which also determines the pseudanthia growth and the ramification and elongation of the axes. With the complete development of the female flowers in the pseudanthia, the branches no longer elongate and bracts (involucral, involucel) are arranged very close to each other in a condensed shape. Therefore there seems to be an association between the shortening of the branches and the elaboration of bracts, which are organized to develop different roles in the pseudanthia, such as protection and attraction.
